# The Fat Controller: Adipocyte Development

**DOI:** 10.1371/journal.pbio.1001436

**Published:** 2012-11-27

**Authors:** Jacqueline M. Stephens

**Affiliations:** Adipocyte Biology Laboratory, Pennington Biomedical Research Center, Louisiana State University, Baton Rouge, Louisiana, United States of America

## Abstract

Obesity is a condition characterized by excess adipose tissue that results from positive energy balance and is the most common metabolic disorder in the industrialized world. The obesity epidemic shows no sign of slowing, and it is increasingly a global problem. Serious clinical problems associated with obesity include an increased risk for type 2 diabetes, atherosclerosis, and cancer. Hence, understanding the origin and development of adipocytes and adipose tissue will be critical to the analysis and treatment of metabolic diseases. Historically, albeit incorrectly, adipocytes were thought to be inert cells whose singular function was lipid storage. It is now known that adipocytes have other critical functions; the most important include sensitivity to insulin and the ability to produce and secrete adipocyte-specific endocrine hormones that regulate energy homeostasis in other tissues. Today, adipocytes are recognized as critical regulators of whole-body metabolism and known to be involved in the pathogenesis of a variety of metabolic diseases. All cells come from other cells and many cells arise from precursor cells. Adipocytes are not created from other adipocytes, but they arise from precursor cells. In the last two decades, scientists have discovered the function of many proteins that influence the ability of precursor cells to become adipocytes. If the expansion of the adipose tissue is the problem, it seems logical that adipocyte development inhibitors could be a viable anti-obesity therapeutic. However, factors that block adipocyte development and limit adipocyte expansion also impair metabolic health. This notion may be counterintuitive, but several lines of evidence support the idea that blocking adipocyte development is unhealthy. For this reason it is clear that we need a better understanding of adipocyte development.

## Introduction

Adipocytes have three primary functions. Fat cells are insulin sensitive, they store lipid, and they secrete hormones that act in distant tissues. Of note, the disruption of any one of these adipocyte functions results in an unhealthy metabolic disease state that increases risk for type 2 diabetes (T2DM). Hormones that are produced exclusively in adipocytes, such as leptin and adiponectin, have various functions including the regulation of food intake and modulation of sensitivity to insulin, a hormone involved in regulating blood glucose levels. Unlike some other hormones, insulin is required for survival and a loss in sensitivity to this hormone is called insulin resistance. Insulin resistance is what defines and characterizes patients with type 2 diabetes. If adipocytes are not insulin sensitive, then metabolic perturbations in other tissues occur. For years, scientists thought that blocking lipid storage or inhibiting fat cell development might be an effective anti-obesity therapy. Common sense suggests that less fat mass would be healthier. Yet, we know from mouse and human studies that this is not necessarily true. When lipid cannot be stored in adipocytes, it accumulates in other tissues where it should not be stored. This condition, known as ectopic lipid, has been associated with insulin resistance and the development of T2DM [1]. All these functions support the critical role adipocytes play in whole body energy balance. Disrupting any of these functions can lead to a metabolic disease state.

## All Adipocytes Are Not Equal

There are different types of adipocytes that are broadly classified into three main types based, in part, on the color of the fat tissue: white, brown, or beige. The overall function of white adipocytes is to store energy, while the function of brown adipocytes is to dissipate energy in a heat-producing process called thermogenesis. The function and origin of beige cells is less clear and under intense investigation. It's thought that they arise from unique precursor cells [2], but there is also evidence that they may arise from white adipocytes in a process referred to as trans-differentiation [3]. They have some properties of brown fat cells, including possibly the ability to dissipate energy, and thus to serve as an anti-obesity therapeutic. Although there are exceptions to the rule, it is thought that an increase in white fat is unhealthy, while an increase in beige and/or brown fat is beneficial. To complicate matters even more, the cellular and lipid composition of adipose tissue varies substantially in different anatomical locations and under different environmental conditions.

## What Adipocyte Development May Tell Us

Adipocytes play an important role in maintaining good health. Obesity is an excess accumulation of adipose tissue, an organ that is largely comprised of fat cells. The expansion of adipose tissue can be associated with hyperplasia, an increase in cell number, or hypertrophy, an increase in cell size. Typically, hypertrophy without hyperplasia leads to adipocytes that are metabolically unhealthy. Activation of biological pathways that favor adipocyte differentiation, the formation of adipocytes from precursor cells, produces an increase in the number of adipocytes. Adipocytes are dynamic cells that have critical functions to maintain whole body energy homeostasis. The resulting adipocyte expansion is critical for insulin sensitivity and overall metabolic health. Therefore, understanding the development of adipocytes is not only interesting in terms of biology, it is also needed to enhance our understanding of the pathogenesis of metabolic diseases including obesity and T2DM.

**Figure 1:**
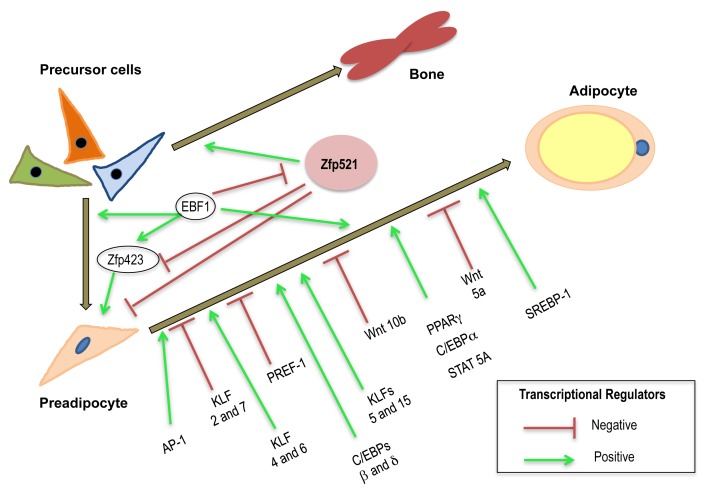
Zfp521 is novel modulator of adipocyte commitment and differentiation. Many transcription factors are induced during adipocyte differentiation. Some of these, like members of the AP-1 family, are induced early during adipocyte development. Other transcription factors like PPARγ promote adipocyte differentiation. Adipocyte development is also influenced by several transcription factor families that have negative effectors on adipogenesis. Zfp521 is a novel modulator of adipocyte development that mediates its inhibitory effects on adipocyte development by regulating the activity of two other transcription factors, Zfp423 and Ebf1.

Studies in the last two decades have revealed new proteins involved in inhibiting or promoting adipocyte development [4],[5]. As indicated in Figure 1, there are many transcriptional regulators that have been identified that promote or inhibit adipogenesis. The development of fully differentiated mature adipocytes from precursor cells is an elegant progression involving the sequential activation of a battery of transcription factors. Early events include the activation of members of the AP-1 family of transcription factors and continue with the induction and expression of PPARγ, a critical pro-adipogenic transcription factor (see Figure 1). Other transcription factors facilitate adipocyte maturation including STATs, members of the KLF family of proteins, SREBP-1, and members of the C/EBP family [5]. There are also potent negative repressors of adipocyte differentiation including Pref-1 and members of the GATA and Wnt families [5]. To date, the majority of studies in adipocyte development have identified transcription factors that contribute to terminal adipocyte differentiation. However, there has been less progress in the identification of factors that contribute to the earlier events that affect both cell fate choice and lineage commitment. Recent progress in this area has identified zinc finger protein 423 (Zfp423) as an early player in adipocyte determination [6]. In this issue of *PLOS Biology*, the Rosen laboratory at the Beth Israel Deaconess Medical Center in Boston has shown Zfp521 also has a significant role in adipose commitment and differentiation [7]. The study demonstrates that overexpression of this protein blocks adipocyte development and that inhibition of Zfp521 promotes fat cell development. Moreover, the results indicate that Zfp521 acts by binding to early B cell factor 1 (Ebf1). Ebf1 is a transcription factor that can inhibit the expression of Zfp423 and is necessary for the generation of adipocyte progenitors and inhibiting Zfp423 expression. Many transcription factors that inhibit adipocyte development can also promote bone development. This is not surprising, considering that certain stem cells can differentiate into a variety of cell types including fat, bone, and muscle [8]. Since Zfp521 can inhibit adipocyte development, it is no surprise that it can also promote bone development [9]. Overall, these original observations have identified a novel pathway, involving at least three transcription factors, which function to control a critical switch in the commitment decision between the adipogenic and osteogenic lineages. Studies to understand how commitment of cells is regulated to become bone or fat are highly relevant in understanding the dual epidemics of osteoporosis and obesity.

## Adipose Tissue Is Important

Adipocytes are needed to store lipid so that the fat is not stored somewhere else (ectopically), a condition that results in a metabolic disease state. It might seem illogical, but the overwhelming amount of data suggests that limiting adipose tissue expansion is unhealthy. Also, most people with T2DM are obese, but most obese people are not diabetic and constitute a large population identified as metabolically healthy obese [10],[11]. Obese people who are healthy have a 38% lower mortality risk [12]. In these people, the primary three functions of adipocytes are intact. Therefore, excess fat is not necessarily bad and it is better to make more adipocytes than to block the formation of new adipocytes (more information in [13]). It is not known whether transcription factors that modulate adipocyte commitment and development are involved in the favorable metabolic profile observed in healthy obese people.

## Future Directions and Challenges

Identification of new players in adipocyte development and obesity is essential. In the past year, many of these studies have described a variety of factors shown to increase beige adipocyte numbers. Most of these studies are conducted in rodents. Every year, the US Centers for Disease Control continues to reveal increases in the incidence of overweight and obesity in America. So, although scientists continue to make substantial progress in understanding adipocyte development, the obesity epidemic continues to rise. Of course, one could argue that the obesity epidemic can be attributed to high-fat diets and to lack of physical activity, which are strongly affected by a range of factors, including socio-economic status. Although there is no question these factors play a role, the complexity of adipocyte development, though critical, is rarely considered. All that we have learned so far indicates that there are many things we still don't understand. Our recent discoveries have told us that fat cells can have multiple origins and that each type (white, fat, and beige) and each location (belly, limbs, etc.) can have different cellular precursors and substantial differences in function. Also, men and women store fat in different places, so gender complicates these studies. Most of the things we know about adipocyte development has come from rodent studies. Rodents are an invaluable model system, but humans store fat in different places and have dramatically different living conditions. Therefore, an obvious challenge will be to determine if our observations in rodents are also representative of human adipocyte biology. Perhaps, the greatest challenge will be to manipulate fat storage in specific locations and provide therapeutics that generate enough beige or brown fat to dissipate energy, but not so much that it affects body temperature. Overcoming these challenges will be relevant in treating metabolic diseases, in particular T2DM. The present study by Rosen and colleagues reveals a new zinc finger protein Zfp 521 in early preadipocyte differentiation. Moreover, the study provides mechanistic information on the ability of Zfp521 to mediate its inhibitory effects on adipocyte development by modulating the activity of two other transcription factors, Zfp423 and Ebf1. These studies reveal the complexity of adipocyte development and provide additional support for the existence of a critical switch in the commitment decision between osteogenic and adipogenic lineages. Follow up studies will be needed to identify the Zfp521 target genes that mediate its anti-adipogenic effects. An obvious question that arises from these studies is whether Zfp521 has the same effect on all preadipocyte precursors including cells destined to become brown, white, or beige adipocytes. Ultimately these studies not only enhance our knowledge of the early events of mesenchymal differentiation, but also may represent a feasible tactic in the struggle to promote healthy bones and favorable metabolic outcomes.

## Abbreviations

Ebf1: early B cell factor 1
T2DM: type 2 diabetes
Zfp423: zinc finger protein 423
